# Changes in physiotherapy students’ knowledge and perceptions of EBP from first year to graduation: a mixed methods study

**DOI:** 10.1186/s12909-018-1212-4

**Published:** 2018-05-11

**Authors:** Maureen P. McEvoy, Lucy K. Lewis, Julie Luker

**Affiliations:** 1 Sansom Institute for Health Research, University of South Australia, Adelaide, Australia; 2 School of Health Sciences, Faculty of Medicine, Nursing and Health Sciences, Flinders University/Health Sciences Building, Repatriation General Hospital, 210 -216 Daws Rd, Daw Park, Adelaide, South Australia 5041 Australia; 3 School of Health Sciences, University of South Australia, GPO Box 2471, Adelaide, South Australia 5001 Australia

**Keywords:** Evidence-based practice, Physiotherapy, Students, Mixed methods, Knowledge, Perceptions, Confidence, Practice, Relevance, Education

## Abstract

**Background:**

Dedicated Evidence-Based Practice (EBP) courses are often included in health professional education programs. It is important to understand the effectiveness of this training. This study investigated EBP outcomes in entry-level physiotherapy students from baseline to completion of all EBP training (graduation).

**Methods:**

Mixed methods with an explanatory sequential design. Physiotherapy students completed two psychometrically–tested health professional EBP instruments at baseline and graduation. The Evidence-Based Practice Profile questionnaire collected self-reported data (Terminology, Confidence, Practice, Relevance, Sympathy), and the Knowledge of Research Evidence Competencies instrument collected objective data (Actual Knowledge). Focus groups with students were conducted at graduation to gain a deeper understanding of the factors impacting changes in students’ EBP knowledge, attitudes, behaviour and competency. Descriptive statistics, paired t-tests, 95% CI and effect sizes (ES) were used to examine changes in outcome scores from baseline to graduation. Transcribed focus group data were analysed following a qualitative descriptive approach with thematic analysis. A second stage of merged data analysis for mixed methods studies was undertaken using side-by-side comparisons to explore quantitatively assessed EBP measures with participants’ personal perceptions.

**Results:**

Data were analysed from 56 participants who completed both instruments at baseline and graduation, and from 21 focus group participants. Large ES were reported across most outcomes: Relevance (ES 2.29, *p* ≤ 0.001), Practice (1.8, *p* ≤ 0.001), Confidence (1.67, *p* ≤ 0.001), Terminology (3.13, *p* ≤ 0.001) and Actual Knowledge (4.3, *p* ≤ 0.001). A medium ES was found for Sympathy (0.49, *p* = 0.008). Qualitative and quantitative findings mostly aligned but for statistical terminology, participants’ self-reported understanding was disparate with focus group reported experiences. Qualitative findings highlighted the importance of providing relevant context and positive role models for students during EBP training.

**Conclusions:**

Following EBP training across an entry-level physiotherapy program, there were qualitative and significant quantitative changes in participants’ knowledge and perceptions of EBP. The qualitative and quantitative findings were mainly well-aligned with the exception of the Terminology domain, where the qualitative findings did not support the strength of the effect reported quantitatively. The findings of this study have implications for the timing and content of EBP curricula in entry-level health professional programs.

**Electronic supplementary material:**

The online version of this article (10.1186/s12909-018-1212-4) contains supplementary material, which is available to authorized users.

## Background

Evidence based practice (EBP) where patient values, clinical findings and best research evidence are integrated in patient decision-making, is now widely accepted as an essential component both in clinical practice and education of entry-level health professionals [1, 2]. The principles of EBP dovetail well with the pedagogical principles applied by educational institutions in health professional education. These include: creating an academic culture that values and understands the relevance of EBP, providing sound training in client and inter-professional communication skills, profession-specific technical skills, and skills in the five steps of EBP (ask, access, appraise, apply, assess) [1]. The challenge has been in designing, teaching and evaluating EBP courses to meet the expectations of accreditation bodies of health professional programs.

The effectiveness of EBP training has been investigated largely in the medical profession as shown in systematic reviews by Coomarasamy and Khan [3] and Ilic and Maloney [4]. A further systematic review by Wong et al. [5] on changes in EBP outcomes after entry-level EBP training, encompassed all entry-level health professions, but found 22 of the 27 included studies were conducted in medicine. Most quantitative studies investigating the effectiveness of EBP training outside medicine have been pre-post in design with short term follow-up after one or two courses, and included students [6, 7] or clinicians [8]. Very few studies have investigated EBP outcomes across a wider time frame in the non-medical health professions; the exceptions were McEvoy et al. [8] who investigated quantitative changes in EBP outcomes in physiotherapy students from graduation to one or 2 years in the workforce and Lewis et al. [9] who evaluated changes in entry-level health professional students’ EBP knowledge, attitudes and behaviours after two sequential EBP courses spanning up to 2 years.

A solely qualitative study on student perceptions of EBP was undertaken by Ilic and Forbes [10] but in recent years there has been an increase in the use of mixed methods studies, integrating both qualitative and quantitative findings. These have included studies in medical students [11], physiotherapy clinicians [12] and concurrent samples of first, second and third year physiotherapy students [13]. However, no previous study has presented quantitative findings of the same students before and after completion of EBP courses undertaken over the duration of a health professional program i.e. matched data comparing first year to graduation, and integrated this with qualitative findings; this is the niche of the current study.

The aim of this study was to investigate and integrate qualitative perceptions, and quantitative changes in physiotherapy student EBP outcomes (self-reported knowledge, attitudes and behaviours, and actual knowledge), from ‘baseline’ (prior to any EBP training) to ‘graduation’ (after completion of all EBP training).

## Methods

### Design

This mixed methods study used an explanatory sequential design where a quantitative sub-study was followed by a qualitative sub-study. Qualitative data were sought to gain a deeper and richer understanding of the factors impacting changes in physiotherapy student knowledge, attitudes, behaviour and competency with EBP. Quantitative and qualitative findings were then merged to satisfy the aims of the study [14]. Ethical approval for the study was gained from the Human Research Ethics Committee of the University of South Australia (protocol numbers 0000021077 for the questionnaire data and 0000030567 for the focus group data). Data were collected in 2010 and 2013.

### Participants

Participants were Bachelor of Physiotherapy (+/− Honours) entry–level students enrolled in 2010 and graduating in 2014 from the University of South Australia. In the standard Bachelor of Physiotherapy program all students completed three mandatory EBP courses in the 1st, 2nd and 4th years of the program. For students undertaking the Bachelor of Physiotherapy program with Honours, the final EBP course was replaced by a ‘Health Sciences Honours Thesis’ course. The EBP curriculum across the three courses is presented in Table 1. A single cohort was invited to participate; as the study investigated changes from baseline (where students had no known EBP training) to graduation (which was the completion of the EBP training), participants were included only if they provided matched data for the two test occasions and were entitled to graduate in 2014.

**Table 1:** Curriculum and assessment for three Evidence-Based Practice courses over a four year program

Course	Evidence Based Practice 1	Evidence Based Practice 2	Evidence Based Practice 3
Duration	13 weeks	13 weeks	16 weeks
Contact hours			
	2 h of face-to-face lectures 12 h of face-to-face tutorials 36 h of online learning modules	26 h of face-to-face lectures/discussions 11 h of tutorials 1 h of computer practical session	1 week intensive workshop (face-to-face lectures, interactive library sessions, workshop practical sessions) group (*n* = 5) work with facilitator to undertake a systematic review
Content			
	• principles and conflicts concerning the best available research evidence • hierarchies of research evidence • methodologies and assumptions underlying quantitative and qualitative research approaches • fundamental skills in accessing electronic databases	• introduction to and application of different CATs • development of research questions in a PICO format • data analysis and presentation • implications and conclusions of findings • establishment of systematic search strategies • selection of appropriate databases • limitations to research	• procedure to undertake and report a systematic review • developing a focussed PICO for a specific topic • developing eligibility criteria • database selection, developing and saving a search strategy • managing references/software • choice and application a CAT • data presentation • interpretation and application of findings
Assessment			
	1 written quiz of 15 questions (15%) 1 open book written test (35%) Final written exam (50%)	1 multiple choice and short answer test (15%) 2 tutorial presentations (2 × 5%) 2 A4-page summary of presentation material (2 × 12.5%) Final written exam (50%)	Group systematic review proposal (15%) Complete and write-up a systematic review (70%) Peer mark (10%) Facilitator mark (5%)

*CAT* Critical appraisal tools, *PICO* Population Intervention Comparison Outcomes

### Procedure

#### Data collection questionnaires

Data were collected using two questionnaires, both administered to participants at baseline and at graduation (two test occasions). The Evidence-Based Practice Profile (EBP^2^) questionnaire measured self-reported knowledge, attitudes and behaviours [15] and the Knowledge of Research Evidence Competencies (K-REC) instrument measured participants’ actual EBP knowledge [16]. The EBP^2^ questionnaire includes 58 five-point Likert scale items that address five EBP domains [Relevance (14 items): the value, emphasis and importance placed on EBP; Terminology (17 items): understanding of common research terms; Confidence (11 items): perception of ability with EBP skills; Practice (9 items): the use of EBP in clinical situations; Sympathy (7 items): perception of the compatibility of EBP with professional work]. The K-REC instrument evaluates Actual Knowledge of EBP using nine items (short answers to clinical scenarios, multiple choice and true/false questions, maximum score of 12). Both instruments were developed specifically for the health professional disciplines, are relatively quick for participants to complete and have demonstrated psychometric properties [15, 16]. The details of both instruments, including the domains, items, and detailed psychometric properties are presented in Table 2.

**Table 2:** Overview of definitions, items and psychometric properties of Evidence Based Practice Profile questionnaire and Knowledge of Research Evidence Competencies instrument

Instrument	Domain definitions	Number items (maximum score)	Psychometric properties
EBP^2^ domains (5 point Likert scale)			
Relevance	Value, emphasis and importance placed on EBP	14 items (70)	Internal consistency (Cronbach’s alpha 0.96) Test-retest reliability (ICC’s 0.77–0.94 over five domains) Convergent validity for three comparable domains with less comprehensive questionnaire [23] (Pearson correlations: Practice 0.66, Confidence 0.80, Sympathy 0.54) Discriminative validity for EBP exposure (ANOVA *p* < 0.0001–0.004).
Terminology	Understanding of common research and statistical terms	17 items (85)	
Sympathy	Compatibility of EBP with professional work	7 items (35)	
Practice	Use of EBP in clinical situations	9 items (45)	
Confidence	Perception of ability with EBP skills	11 items (55)	
K-REC (short answers to clinical scenario, MC, T/F questions)			
Actual Knowledge	Actual knowledge of EBP	9 items (12)	Construct validity (*p* < 0.0001 comparing scores for EBP exposed and non-exposed participants) Responsive validity for impact of EBP training (*p* < 0.001, effect size 1.13) Test-retest reliability for individual item and total scores (Cohen’s kappa and ICC range 0.62 to perfect agreement Inter-rater reliability for individual item and total scores (0.83-perfect agreement)

*EBP* Evidence Based Practice, *EBP*^*2*^ Evidence Based Practice Profile, *K-REC* Knowledge of Research Evidence Competencies, *MC* multiple choice, *T/F* True/False

#### Focus groups

All graduating students who had completed the final EBP questionnaires and were eligible for graduation were invited by email to participate in semi-structured focus groups. Students who responded were provided with additional information, and written informed consent was obtained. The focus groups were conducted on the university campus by an experienced facilitator who had not been involved in participants’ EBP teaching (JL). A semi-structured question guide used by the facilitator ensured all areas were covered in each group (Additional file 1) but did not limit the flow of relevant information that arose during group discussions. The research team had developed the question guide to cover areas relevant to the study aims including gaining the perspective of final year students on EBP training across and at completion of the physiotherapy program, and perceptions of future use of EBP in the first year in the workplace. Focus group discussions explored students’ perceptions of EBP training (across the three courses), including knowledge and skill development, value and relevance of EBP, EBP behaviours, current use of EBP and role models during the program. Sessions were digitally audio-recorded. An AUD25 honorarium was paid to each focus group participant.

### Data management and analysis

#### Quantitative analysis

De-identified data were entered into Predictive Analytic Software (PASW) Statistics 17.0 (Chicago, IL). Participants were only included if matched data were available for the two test occasions. For the EBP^2^ questionnaire, only domain data where participants had completed 100% of domain items, for that particular domain, on both the first and last occasions of testing were included for analysis. For the K-REC, participants were required to have completed at least 70% of items on both test occasions for inclusion in the analysis. A less stringent inclusion criteria was applied for the K-REC as participants with no knowledge of EBP at baseline were considered more likely to omit items. For the EBP^2^ questionnaire the Likert scores were treated as interval data; maximum domain scores varied (Relevance 70, Terminology 85, Confidence 55, Practice 45, Sympathy 35), due to the different number of items per domain. The K-REC instrument for Actual Knowledge was scored according to set scoring guidelines, with a maximum score of 12. Descriptive statistics were calculated for each of the five EBP^2^ domain scores, K-REC Actual Knowledge domain total score and demographic information for the test occasions. Paired t-tests, 95% CI and effect sizes were used to examine the changes in domain scores between the two test occasions. Alpha levels of less than 0.05 were considered statistically significant. Effect sizes (ES) were classified as small (≤ 0.20), medium (0.50) and large (0.80) [17].

#### Qualitative analysis

Digitally audio recorded focus group sessions were transcribed verbatim. Transcribed data were de-identified and entered onto NVivo 10 software (QRS International Pty Ltd) for coding and data management. Data analysis followed a qualitative descriptive approach with thematic analysis as described by Stanley [18]. Information relevant to the study aims was inductively coded and thematic development was conducted in stages. Each analytical stage involved independent consideration, discussions and consensus by two researchers (LB, JL), who were naïve to the quantitative results. Initially, codes were inductively derived from these data in an iterative process of attributing codes to small sections of meaning, moving back and forward across focus groups and constantly comparing data and codes. Codes were then grouped into logical and meaningful clusters in a hierarchical tree structure, forming descriptive themes and sub-themes. These themes were described, along with illustrative quotes from the focus groups.

#### Merged analysis

A second stage of merged data analysis for mixed methods studies was undertaken using side-by-side comparisons as described by Creswell and Plano Clark [14]. This explored the alignment of quantitatively assessed measures of participants’ EBP development with their personal perceptions. Using this technique, the tabulating of quantitative alongside qualitative results became the means for merging these data. It enabled the comparison and communication of congruent and divergent interpretations, and facilitated the drawing of conclusions.

## Results

### Participants

There were 127 physiotherapy students enrolled in the first EBP course and 96 (77.2%) completed the initial questionnaire; there were 125 students enrolled in the third EBP or Honours course and 109 (87.2%) completed the final questionnaire. Overall, there were 56 physiotherapy students who provided matched data by completing both the initial and final questionnaires. Baseline characteristics for the 56 participants: gender 41F/15M, age [mean (SD) range in years] 19.1 (3.6), 17–44 years, English was first language for *n* = 45, not first language for *n* = 8 and not reported for *n* = 3. The baseline characteristics of the students who completed the final questionnaires but were not included in the matched dataset were: gender 40F/29M, age [mean (SD) range in years] 19.8 (4.5) 17–46 years. There were 21 participants involved across four focus groups conducted in November 2013 (*n* = 6, 5, 6 and 4 participants in the respective groups*).* Baseline characteristics for the 21 focus group participants: gender 16F/5M, age [mean (SD) range in years] 18.9 (1.9), 17–25 years, English was first language for *n* = 16, not first language for *n* = 5. No time limit was imposed but focus groups lasted an average of 46 min (range 43–51 min).

### Quantitative findings

While there were 56 participants with matched data from baseline to ‘graduation’, there was not complete matched data from all 56 participants for each of the six domains. The number of matched sets of data for the individual domains were: Relevance, Confidence and Sympathy *n* = 55 each, Terminology *n* = 53, Practice *n* = 51. For the K-REC instrument (Actual Knowledge) 50 participants completed at least 70% of the items on both occasions. Changes in scores for each domain (mean, 95% CI, *p*-value and ES) from baseline to ‘graduation’ are presented in Table 3.

**Table 3:** Descriptive data and analyses for six EBP domains for matched groups from baseline to ‘graduation’

Domain	Baseline mean (SD) [95% CI]	Graduation mean (SD) [95% CI]	Baseline→ Graduation Mean increase (95% CI)	Baseline→ Graduation Raw *p* values	Effect size (ES)
Relevance (*n* = 55)	51.1 (7.0)	64.7 (4.5)	13.6 (11.7–15.5)	*p* ≤ 0.001	ES 2.29↑
Terminology *n* = 53)	36.8 (11.1)	68.2 (8.6)	31.3 (27.7–35.0)	*p* ≤ 0.001	ES 3.13↑
Confidence (*n* = 55)	33.7 (7.6)	44.9 (5.8)	11.3 (9.1–13.4)	*p* ≤ 0.001	ES 1.67↑
Practice (*n* = 51)	17.5 (5.2)	27.2 (5.5) (26.9) (5.4))	9.7 (7.7–11.6)	*p* ≤ 0.001	ES 1.8↑
Sympathy(*n* = 55)	21.6 (2.7)	23.5 (4.5)	1.8 (0.5–3.2)	*p* = 0.008	ES 0.49↑
Knowledge (actual) (*n* = 50)	2.6 (1.3)	8.8 (1.6)	6.2 (5.6–6.8)	*p* ≤ 0.001	ES 4.3↑

*CI* Confidence interval, *EBP* Evidence-based practice

**Fig. 1:**
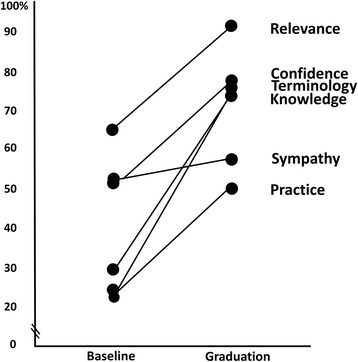
Change from baseline to graduation as percentage of possible maximum score for each EBP domain

Maximum domain scores varied (Relevance 70, Terminology 85, Confidence 55, Practice 45, Sympathy 35, Actual Knowledge 12) due to the differences in the number of items in each domain. Figure 1 presents the domain scores as a percentage of the possible maximum (100%) score from baseline to ‘graduation’.

### Qualitative results

Four major descriptive themes with sub-themes were found that reflected the experiences and perceptions of students over the years of EBP education (see Table 4). These were: 1) a shift in thinking over time; 2) the need for relevance and context; 3) learning by doing; and 4) getting the timing right. Participant quotes that support these findings are presented in Additional file 2.

**Table 4:** Descriptive themes and sub-themes reflecting experiences and perceptions of EBP education

	Major descriptive themes	Sub-themes
1	Shift in thinking over time	
		*Role of research not appreciated in clinical practice*
		*Change in perception and confidence by graduation*
2	Need for relevance and context	
		*Rote learning with minimal carry over*
		*Learning preferences*
3	Learning by doing	
		*Role models and reinforcement*
4	Getting the timing right	
		*Optimising clinical placement experiences*
		*Evidence searching skills needed early*

#### 1. Shift in thinking over time

Participants described a substantial shift in their understanding and perceptions of EBP over the 4 years of their program. They were initially almost completely naïve about the relevance of EBP to physiotherapy. Participants described the first 2 years of EBP education as difficult, uninteresting and irrelevant. In the second half of their program, students became increasingly aware that EBP played a major role in many other courses within their program, and also that *‘…it is a big thing’* in their future physiotherapy profession. By the time of graduation, their perceptions of EBP had become very positive. *“I think when it’s your third and fourth year you realise ‘wow this is really important and it’s interesting and it guides our practice and it’s really helpful.’”*

At the commencement of their program, *the role of research in clinical practice was not appreciated* by students. Some students described their expectations of physiotherapy as a static practice, with established treatment practices and protocols. Research as a component of the program came as a shock to many students. *“I thought it was something separate, so physios actually do the work … it’s not really aligned with research.”*

Students reported a *change in perception and confidence by graduation* and that they possessed the knowledge and skills they would need in the workplace. *“I’m pretty confident that I can now search for it well, that I can go through all the databases and know what I have to use for search terms and that I can do a fairly good appraisal, and that I can apply it to a patient.”*

Participants varied in their understanding of some EBP terminology, particularly statistical terms used in manuscripts, however this did not appear to negatively affect their confidence. *“…some of the statistical terminology that you look at and go… I don’t know what that means. But I do feel equipped to find that information myself …”.*

*“…sometimes reading articles, you know like the numbers in brackets and some parts that I still don’t quite get… but I think basically I’ve got the gist of it; I can do it.”*

#### 2. Need for relevance and context

In the first 2 years of the program participants wanted more context to understand the role and relevance of EBP, both within the entire program, and within the profession they were entering. *“Like at the beginning it just feels like a big burden, what’s the point and why are we doing this?”*

In first and second year, physiotherapy students studied EBP with students from other health professional programs. Although they appreciated meeting students from other disciplines, they were disappointed when general examples were provided in lectures and assignments which lacked physiotherapy context. Later in the program, participants grew to appreciate the importance of EBP for their work, but they continued to want greater clinical context and relevance to physiotherapy. Examples were given of a systematic review assignment undertaken during clinical placements. Students were critical of the assignment’s strong emphasis on procedural writing and the poor relationship to the needs of students’ clinical placement patients. *“It was all about how you wrote it and what you included in your writing”. “It would’ve been a lot more helpful for me to be doing more … writing about my patients and researching things for them which I would do in the clinical world…”.*

Early in the program, an inability to see the relevance of EBP to their future work impacted on students’ learning strategies. Students described rote-learning for first and second year examinations, *with minimal carry over* to long-term knowledge retention. Carry over learning into third year was described as a vague familiarity with some words and concepts. *“We rote learnt it... then out of my head again, … I was just like I just want to get this so I get a good mark then leave.”*

Participants expressed their *learning preferences* for EBP, and considered the on-line lectures delivered in first year to be less effective than face-to-face lectures and tutorials. *“I remember everyone was complaining about it, because as first years we couldn’t do our own learning. We didn’t know how... I think that’s why when we got to second year, we actually didn’t know anything.”*

The repetition of content between first year and second year EBP courses was regarded negatively by several participants. Conversely, some viewed repetition more positively and admitted it provided an opportunity to ‘catch up’ and consolidate their learning.

Later in the program, students worked in groups to conduct a systematic review. Many students found the group assignment disadvantageous, as tasks for the review were shared out amongst the group and some felt they missed out on practising important skills.“ *I suppose I don’t feel as confident as probably other people do, because I did miss out on implementing the search strategy actually in the databases and learning properly how to do it.”*

#### 3. Learning by doing

The third year of EBP education was a turning point for participants.*“EBP3 has been the best…We’ve learned the most and how to actually apply it.”*

At this time students learned how to conduct database searches, and how to appraise and select the best evidence to answer their clinical questions. Students reported that these processes were easier to understand when a demonstration lecture was followed by a practical session.

Participants also associated the greater understanding and appreciation of EBP with the commencement of clinical placements in third year, where they could apply EBP principles practically to their own patients. *“…once you start going on placement and your supervisors ask you ‘what kind of evidence is behind this treatment?’ you see the link.”*

Some students distinguished between third and fourth year, highlighting their final year as the point where they commenced customising the research evidence specifically to their individual patients. *“… this year [4*^*th*^*] it’s very specific to your patients and making sure you look at the patient perspective and say, this is the evidence, but is it relevant to them?”*

Participants reported another effective way of ‘learning by doing’ was through conducting their own systematic reviews, either as group projects or for an individual patient’s needs. *“It was helpful in the sense that I have a way better understanding of how to search and also how to critically appraise things. So when I get an article myself, I can look at it and say, ‘no, this is bad methodology’.”*

Having *role models and reinforcement* helped students consolidate their understanding of EBP. Students recalled university staff referring to evidence in lectures and tutorials of non-EBP courses, which reinforced its importance. *“I think they definitely push it, it is a big thing.”*

Clinical placements provided important reinforcement of EBP principles, and students provided many examples of personally using their new EBP skills during placements. These placements also afforded some positive role models for the use of EBP in the workplace. *“Last placement, my tutor often referred to “new evidence in this” to his patients …would explain it to them”.* Students witnessed research evidence being circulated in clinical staff development sessions, and also evidence provided to patients by clinical tutors.

Although participants sometimes witnessed negative EBP role models during clinical placements, such as staff who continued to use disproven interventions, they tended to be disparaging of this non-EBP approach and saw it as outdated.

#### 4. Getting the timing right

There were certain optimal times when participants wanted to receive particular EBP training or information. The current timing of the EBP education had not always met their needs. For example, many believed they would have benefitted from a larger view of the relevance and importance of EBP at the outset of their physiotherapy program*. “I didn’t realise …I was just like, ‘EBP just get through it’ … so maybe if I knew it was going to be such a big part, maybe I would’ve taken it bit more seriously”.*

*Advanced evidence searching and management skills were needed early* in the program*.* These had been taught in the final year, and were then reinforced by practical library tutorials and were seen as highly valuable by the students. Several students said that they would have appreciated learning these searching skills earlier in the program.

*“Just one library session and it changed everything for us. It was like all those times I spent hours researching for an essay, still only got a credit because my resources weren’t good, and if they put that earlier on I think it would help....”*

*“…teaching how to use Endnote properly as well, ‘cause that was particularly good…”*

The skills learned in the final year (i.e. refining database searching, critical appraisal) were considered to be very important for *optimising clinical placement experiences*. Students who attended clinical placements prior to learning these skills felt disadvantaged by this poor timing. Further to this, some participants felt that the perceptions of irrelevance experienced during their first 2 years of EBP education would have been lessened if it had followed a clinical placement experience *“…would definitely appreciate it more in second year after a placement.”*

### Merged data results

The findings from this analytical work are summarised in Table 5. The side-by-side comparison table compared the quantitative questionnaire data with focus group data on eight major findings. These were drawn from the key findings of the quantitative and qualitative research, thus summarising evidence from each sub-study.

**Table 5:** Side-by-side comparison of summarised information from the Evidence Based Practice (EBP) quantitative and qualitative components

Findings	Quantitative questionnaires	Qualitative focus groups	Congruence
Relevance: the value, emphasis and importance placed on EBP	Students rating of EBP relevance increased over the 4 year program. Mean increase 13.6 (95% CI: 11.7–15.5) Raw *p* value *p ≤* 0.001 ES 2.29 increase	The perceived relevance of EBP increased over the 4 year program • Initial experience of EBP education as difficult, uninteresting and irrelevant “*what’s the point and why are we doing this.”* • By graduation EBP was considered highly important *“…really important and it’s interesting and it guides our practice and it’s really helpful.”*	congruent
Terminology: understanding of common research terms	Students rating of their understanding of terminology increased over the 3 years. Mean increase 31.3 (95% CI: 27.7–35.0) Raw *p* value *p ≤* 0.001 ES 3.13 increase	While participants perceived an increase in understanding general research principles over the program, statistical terminology remained a problem for many • Rote learning early in the course was not initially retained • Ongoing poor understanding of statistical terminology. *“I’m not that confident that I know what all the numbers mean in the result section. Sometimes I look at that and think, ‘I’ll just skip over that’…”*	divergent for statistical terminology
Confidence: perception of ability with EBP skills	Students rating of their confidence increased over the 3 years. Mean increase 11.3 (95% CI: 9.1–13.4) Raw *p* value *p ≤* 0.001 ES 1.67 increase	Participants perceived increased confidence in EBP skills over the program • Initially had no awareness or skills regarding research within physiotherapy • By graduation they were confident of possessing the knowledge and skills they would need in the work place	congruent
Practice: the use of EBP in clinical situations	Students rating of their understanding of practice use increased over the 3 years. Mean increase 9.7 (95% CI: 7.7–11.6) Raw *p* value *p ≤* 0.001 ES 1.8 increase	Participants reported a big increase in their understanding of EBP use in practice • Initially naïve regarding EBP in physiotherapy • By graduation they were unanimous that EBP was essential to their clinical work	congruent
Sympathy: perception of the compatibility of EBP with professional work	Students rating of sympathy increased over the 3 years. Mean increase 1.8 (95% CI: 0.5–3.2) Raw *p* value *p* = 0.008 ES 0.49 increase	Participants’ understanding of the compatibility of EBP with professional work increase greatly over the program • No initial appreciation of EBP or research within physiotherapy • *“…it’s such a big part of our degree that you can’t go into practice and not do it now”* • Some uncertainly expressed about how EBP would fit with busy day-to-day professional work	congruent
Actual Knowledge: measure on a knowledge test	Students’ actual knowledge increased over the 3 years. Mean increase 6.2 (95% CI: 5.6–6.8) Raw *p* value *p ≤* 0.001 ES 4.3 increase	No data available	only quantitative results available
Need for relevance and context	No data available	Participants understanding and appreciation of EBP accelerated in the final years, once it became more clinically relevant to them • *Learning by doing* –understanding accelerated once clinical placements commenced • Need more clinical examples earlier in the EBP course • Need an earlier appreciation of the context and relevance of EBP to physiotherapy practice • Undertaking SRs assisted context and learning • Positive EBP role models reinforced learning	only qualitative results available
Getting the timing right	No data available	Participants identified optimal times to receive particular EBP training or information • Evidence searching, appraisal and Endnote skills are needed early in the program • All skills needed prior to clinical placements	only qualitative results available

*CI* confidence interval, *ES* effect size

#### Congruent findings

The quantitative and qualitative findings were congruent regarding participants’ perceptions of improvements in several domains of EBP learning over the 4 year program for several EBP domains (Relevance, Confidence, Practice, Sympathy).

#### Divergent findings

The quantitative and qualitative components of this research provided different perspectives on only one aspect of EBP learning. While the questionnaires demonstrated a large increase in participants’ understanding of research terminology (ES 3.13), during the focus groups some participants declared poor understanding of statistical terminology.

#### Unique findings

There was a significant increase in Actual EBP knowledge measured by the K-REC questionnaire over the program (*p =* 0.001; ES 4.3). Data on Actual Knowledge were not collected qualitatively, however the increase is reflected in qualitative findings of perceived increased understanding, appreciation and confidence in EBP.

The focus groups were able to uncover some unique information that was unavailable in the quantitative questionnaires. Participants declared strongly that their EBP learning experience was dependent on the how they perceived *relevance and context* of the topic. Early in their program, participants could not see how the EBP information could be relevant to their future physiotherapy practice, and found the courses arduous. An important positive change in the perception and understanding of EBP followed the commencement of clinical placements in third year. Valuable information for educators was provided on *getting the timing right* i.e. the importance of developing EBP skills and knowledge at the optimal time in their 4 year program.

## Discussion

This study found that there were significant changes with mostly large effect sizes for the quantitatively assessed domains of Relevance, Terminology, Practice, Confidence, Sympathy and Actual Knowledge in physiotherapy students from first year to graduation. These findings mostly coincided with the qualitative findings but were disparate for ‘statistical terminology’ where participant perceptions of large changes in their self-reported understanding of terms was not matched by focus group-reported experiences. Additional themes that emerged in the qualitative data were the need for relevance and context, and the importance of getting the timing right for EBP training in entry-level health professional education.

A mixed methods pre-post study was undertaken over a period of 7 months by Bozzolan et al. [13] in three concurrent courses in three different groups of 1st (*n* = 26), 2nd (*n* = 28), and 3rd (*n* = 19) year physiotherapy students. Scores for EBP knowledge, attitudes, skills, practice and competency were substantially different before the first course and after the final EBP course. This supports findings in the current study where there were significant changes in matched student data after three courses. However, interestingly in Bozzolan et al. [13] the scores changed significantly within the 1st and 2nd year students but not within the 3rd year students. As reported in the current study, the qualitative analysis in Bozzolan et al. [13] revealed that students valued EBP but recognised the barriers and observed variations in the support and role-modelling provided by clinicians. Further support for the current study findings were found by McEvoy et al. [19]; significant differences in self-reported EBP domains (Relevance, Terminology, Confidence, Sympathy and Practice) were reported in a cross-sectional study of 914 health professional students across five disciplines, based on exposure to EBP training (*p* < 0.003) and stage of training (year level of professional program) (*p* < 0.001).

More recently, Ilic and co-authors have conducted a series of quantitative and qualitative studies researching medical students’ perceptions of the importance of EBP [10] and approaches to teaching beyond a didactic presentation style [11, 20]. The qualitative findings suggested that medical students recognise the importance of Evidence-Based Medicine principles and application in their training and future clinical practice [10].

As indicated in the curriculum (Table 1) the current study used a multifaceted approach with a combination of face-to-face lectures, workshops, practical training, hand-on experiences, on-line library skills training progressing to more sophisticated searching, and individual to more independent group work. The assessments were similarly scaffolded from written quizzes and exams to tutorial presentations toward undertaking a systematic review as a group under the guidance of a facilitator. An overview of systematic reviews into teaching of EBP supports undergraduate training strategies that focus on multifaceted, clinically integrated approaches [21].

While the formal EBP curriculum delivered over the three EBP course in the current study, were multifaceted, the included topics may be considered as ‘classic’ EBP within the taxonomy developed by Shaughnessy et al. [22]. Further levels of training involving ‘information mastery’ where EBP principles are incorporated into patient care with reflection on clinical outcomes and integration with patient values, will be developed with further clinical experience. However, in the qualitative component of the current study participants alluded to experiencing some initial progression toward these later stages presented in the taxonomy, in final year clinical placements, which is promising.

The lack of congruency between data gained through questionnaires and focus groups about statistical terminology knowledge may initially appear surprising; that students acknowledged, or felt ‘safe’ to reveal their inadequacies with statistical terminology in the focus groups amongst peers, adds validity to this method of data collection for EBP knowledge, attitudes and behaviours. However, it was not surprising that the lack of knowledge seemed to be more about the *application* of the terminology in reading and interpreting the results sections of research studies, which was the element teased out in the focus groups. Interestingly, while aware of a lack of skill in more immediate application, some students commented on their ability to know where to look for the information.

Students need context for EBP and this may not be apparent in the early years of the program where many courses focus on the foundational sciences (anatomy, physiology, biomechanics). With progression to courses involving clinical application, the appreciation of, and commitment to EBP grows. All possible opportunities, particularly early in the program to provide realistic examples of EBP in practice within the profession, and in practical activities that support the theory of EBP, are perceived as important to students. Associated with this is determining the optimal timing of formal EBP training in three to 4 year physiotherapy (and other) programs, to achieve the most favourable outcomes for EBP domains. While students value early exposure to EBP, they are unsure about whether this should commence in the format of formal EBP courses, as early as the first year of University study. The students indicated that they struggled to understand the relevance and context for EBP in the early years of training. It is possible that 1st and 2nd year students find it difficult to comprehend how EBP principles might be integrated into practice, when they have had limited exposure to clinical placements, and an evidence-based approach to clinical decision making in the ‘real world’.

Despite this difficulty with EBP training in the early years, it is clear that reinforcement of EBP skills at many levels is needed for students. For example, repetition of database searching with a demonstration of the skills, followed by practical application, are perceived to enhance abilities. Role-modelling by lecturers and clinical educators, who introduce the language and examples of EBP is observed by students, as is the lack of this in other teachers. This modelling may occur in clinical courses, which students perceive to provide more meaningful experiences for application and assessment of EBP skills to complement formal EBP courses. Questions about the value of stand-alone versus integrated EBP courses and/or whether clinical courses are places for reinforcement of EBP skills, are ongoing. As EBP educators we need to explicitly identify and recognise the informal EBP training that occurs in other courses. It may be, that with maturity in our understanding of EBP and the importance of EBP as an underpinning concept, there is a shift toward greater immersion of EBP in the teaching of all courses.

The study is not without limitations; the findings are generalizable only to physiotherapy students in an Australian University and the lack of a control group may have contributed to maturation bias. However, the strength of the current study was in being the first to use matched data to explore changes in students’ knowledge, attitudes and behaviours following a series of EBP courses across an entire entry-level program, using both matched quantitative, and qualitative data, rather than a focus on comparing singular approaches. The combined mixed methods approach of the current study has allowed the disentanglement of elements of self–reported EBP domains of Terminology, Relevance, Sympathy, Confidence and Actual Knowledge, which may contribute to changes in practice.

## Conclusions

Completion of EBP courses over a 4 year entry-level physiotherapy program resulted in significant changes, with large effect sizes (1.7–4.3) for five of six domains relating to EBP: Relevance, Confidence, Terminology, Actual Knowledge and Practice. For the domain of Sympathy, the ES was medium (0.5). The qualitative and quantitative findings were mainly well-aligned with the exception of the Terminology domain, where the qualitative findings did not support the strength of the effect reported quantitatively. The findings of this study have implications for the timing and content of EBP curricula in entry-level health professional programs.

## Additional files

Additional file 1: Semi-structured question guide for focus groups. Objectives of focus group questions and guide to focus group questions (DOCX 13 kb)

Additional file 2: Qualitative participant data supporting the themes. Examples of supporting quotations for the themes and sub-themes of the qualitative data (DOCX 21 kb)

## Abbreviations

EBP: Evidence-Based Practice
EBP^2^: Evidence-Based Practice Profile
ES: Effect size
K-REC: Knowledge of Research Evidence Competencies
PASW: Predictive Analytic Software
