# Supplementary material for: Changes in physiotherapy students’ knowledge and perceptions of EBP from first year to graduation: a mixed methods study
Source: BMC Med Educ. 2018 May 11;18:109. doi: 10.1186/s12909-018-1212-4 (PMC5948852; doi:10.1186/s12909-018-1212-4)
Supplement: Supplementary file 1 — Semi-structured question guide for focus groups. Objectives of focus group questions and guide to focus group questions (DOCX 13 kb) [file 12909_2018_1212_MOESM1_ESM.docx]

Additional file 1 Semi-structured question guide for focus groups.

Objectives

1. Get perspective of final year students on EBP/research training

2. Get perceptions about future use of, and experience with EBP, in the first year in the workplace

Types of questions to address three area

**Past**: Experience of EBP training during undergraduate program- good aspects and areas for improvement

**Present**: Position now toward or at end of program, in regards to EBP (eg. value /relevance, confidence, knowledge, practice, feasibility)

**Future**: Perceptions of usefulness in the next year in the workplace and practicalities of using an EBP approach in relation to resources (eg. issues with others in the workplace).

*Guide to some of questions that might be useful*

Introductory

How do you see EBP? What is it to you?

Past

What do you think about your training in EBP?

Prompts: You've done EBP1, EBP2, EBP3 (or for Hons, Hons training courses); what was good about these courses? What would make them better/how could they be improved?

How useful has EBP been so far?

Prompts: Has it been used in other courses apart from these (listed above)? Have you seen it used by teachers/clinicians in clinical placements?

Present

How are you feeling about EBP now at or toward the end of your program?

Prompts: How useful is EBP? Have you been able to use an EBP approach?

How do you feel about your knowledge of EBP?

Prompts: How prepared are you to use EBP ie confidence with EBP?

Is it possible to ‘fit in’ the use of an EBP approach/is it feasible, in the management of patients?

Future

How useful will EBP be? (Theoretical)

Do you think you'll be able to use an EBP approach? (Practical)

What resources do you think you'll need, and thoughts about availability?

Do you foresee any other issues with others around you supporting/encouraging or creating barriers to EBP? (Prompts: patients, managers, other physios)-
