# Supplementary material for: Changes in physiotherapy students’ knowledge and perceptions of EBP from first year to graduation: a mixed methods study
Source: BMC Med Educ. 2018 May 11;18:109. doi: 10.1186/s12909-018-1212-4 (PMC5948852; doi:10.1186/s12909-018-1212-4)
Supplement: Supplementary file 2 — Qualitative participant data supporting the themes. Examples of supporting quotations for the themes and sub-themes of the qualitative data (DOCX 21 kb) [file 12909_2018_1212_MOESM2_ESM.docx]

Additional File 2 Qualitative participant data supporting the themes

|  | **Themes and *Sub-themes*** | **Examples of supporting quotations** |
| --- | --- | --- |
| 1 | Shift in thinking over time | Group 1: I don’t know in first year how relevant it is either. First year, I think it was really hard to see the point because it’s such a general …. the second year, yes, and then you have your first placement and then you go on your placement and maybe they talk to you about evidence and stuff and there’s more point.  I think more so in the final year though, because the evidence thing I did in like in third year, but in fourth year, the whole “take into account your patients and take into account the evidence”.  ‘… so like taking articles and saying that this is relevant to what I’m looking at, but not really analysing it, whereas this year it’s very specific to your patients and making sure you look at patient perspective and say, “this is the evidence, but is it relevant to them?”’  ‘..and particularly in the last year, we are sort of a little bit more autonomous…you can use evidence more to tailor for your patients’  ‘There’s such a big place for it [EBP]’ ‘…it is a big thing’  Group 2: ‘I think it’s made more sense to us in the last one or two years probably.’  Group 3: ‘Like at the beginning it just feels like a big burden, “What’s the point and why are we doing this” But as we’ve developed and we’ve learnt practical skills you can apply it to what you’re doing, which makes it more relevant.’  ‘I think when it’s your third and fourth year you realise “Wow this is really important and it’s interesting and it guides our practice and it’s really helpful.”’ |
|  | *Role of research not appreciated in clinical practice* | **Q:** **When you started the degree how important did you feel research was going to be to your career?**  Group 1: ‘Not very.’  ‘I don’t know, I wouldn’t have held it as a priority. Didn’t even think about it probably.’  Group 2: ‘I don’t think I thought about it at all… mainly just anatomy, how the body works, techniques all that kind of stuff.’  ‘I think we all probably just had like a more general idea of what physio was and didn’t know that in the detail like research was quite a big part of it.’  Group 3: ‘I didn’t think it’d be that important.’  ‘I had no understanding that research was involved.’  ‘I thought we’d just need to do the research for like assignments, but definitely not like for patients.’  *‘I thought about doing studies for things … I wanted to be able to do more research in injuries…That’s part of the reason why I started physio. So I had that idea, but I had no idea that the process of the evidence I had no idea we had to actually research each-- get evidence for each treatment and that sort of thing.’*  Group 4: *‘I didn’t think about it’*  *‘I thought it was something separate, so physios actually do the work and then it's not really aligned with research; initially yeah.’* |
|  | *Change in perception and confidence by graduation* | Group 1: *‘I think now after doing EBP, I sort of see there’s a whole different raft of things you can do for any different number of problems and going out there and looking at the research, can see what might be more suitable for your patient.’*  ‘*I’ve got a grasp on it and I could use it’*  *‘I feel confident that I can find it [evidence] and have a good read. I’m not that confident that I know what all the numbers mean in the result section. Sometimes I look at that and think, “I’ll just skip over that”.’*  *‘I think it has been good and it’s been relevant, but yeah, definitely now at the end, I think we’re all feeling a bit more confident in using it.’*  *‘I’m confident in searching and finding and looking at the results and stuff…I probably wouldn’t have the confidence to do an in depth appraisal on it. But I think I could do a good enough job if I was relaying it to my patient.’*  *‘…I feel pretty well prepared to be able to find something that I was looking for, be able to search the relevant databases, but …the numbers and P values and some other things like that, you go, “I’m not really sure about that” and sort of skip to discussion.’*  *‘I think I feel confident with looking up of the evidence and looking at the patient’s values, but I think the whole clinical expertise thing, I think that’ll just come with more practice’*  Group 2: *‘Pretty confident.’ ‘I feel good.’ ‘I feel confident.’*  *‘…so you look it up and you know at least you can trust it if you know how to use trusted sources. It’s not just Googling something.’*  *‘…you know it as the three pillars – I still kind of think when you say evidence based practice I do tend to more think research then your own clinical experience. Obviously all of us we don’t really have much clinical experience...’*  Group 3: *‘I think because of EBP I understand. But when it comes to…some of the terminology, some of the statistical terminology that you look at and go I’d have to Google that, I don’t know what that means. But I do feel equipped to find that information myself or know who to go to to find it out. So I don’t feel like I know everything, but I feel like I know how to find it out.’*  Group 4: *‘I think like we've learned the basics of how to do-- like obviously we need to do research to practice EBP and we've learned how to do that and how to look at an article to see if it's considered good or not, to use.’*  *‘I personally feel that I'm confident enough that if there's something that I don't know, I know where to go to find out, at least on a basic level’*  *‘…sometimes reading articles, you know like the numbers in brackets and some parts that I still don't quite get… but I think basically I've got the gist of it. I can do it.’* |
| 2 | Need for relevance and context | Group 1: *‘I don’t know in first year how relevant it is either. First year, I think it was really hard to see the point’*  ‘*But first year and stuff, it was like, “I don’t care what a P value is”, because I didn’t see the relevance in any of it*.’  *‘…using a P value for what a P value is, not just learning the definition, because that doesn’t mean anything.’*  *‘So we’re learning anatomy and physiology and we’re all like, “I can see how this is going to feed into my career, that makes sense”. Learning the P values and statistics of things that we’ve never read articles before, we didn’t even know what that meant.’*  *‘I think if EBP was more practical in the first two years, it definitely would’ve sunk in a lot quicker perhaps.’*  *‘I think in first and second year, we were mixed with the other Allied Health… I remember one group doing an assignment on something like Med Radiology and we were physios …it wasn’t relevant.’*  Group 2*: ‘When its first year your kind of don’t see the relevance of it.’*  *‘…it’s made more sense to us in the last one or two years probably. The first two years we don’t really have clinical placements so we don’t see the link as much but once you start going on placement and your supervisors ask you what kind of evidence is behind this treatment you see the link.’*  *‘I think yeah because you have that experience of doing it in placement it make – I guess some people learn better by doing things. That’s why I feel more confident to do it because I had the chance to do it in the placements.’*  Group 3: *‘…I remember sitting in the statistics lecture and I absolutely had no idea what the guy was talking about, so lost. I don’t know… I know that I need to be able to understand the basic stuff, but it was just mind blowing. I’m like why are we learning this?’*  *‘Like at the beginning it just feels like a big burden, what’s the point and why are we doing this?’*  *‘…we all want to be good physios and then we’re all interested, obviously, in the human body. So giving us an article about dolphin training isn’t going to attract everyone’s attention.’*  Group 4: *‘…t was never really put into a context of why we actually need to learn it. And I think that's sort of why I didn't really take that much away from the first couple of years until this year it made a lot more sense.’* |
|  | *Rote learning with minimal carry over* | Group 2: *‘It’s a bit like doing year 12 and you have to do maths when you don’t want to, and you just want to be a physio.’*  *‘I was a bit I’ll just learn it before the night.’*  *‘Learning it for the exam and then you don’t really care after that.’*  ‘*We rote learnt it…. so we just went right, bias, bam, bam, bam, retained it then splurged it out on the exam or whatever we had and that was it. Then out of my head again, went straight out…I was just like I just want to get this so I get a good mark then leave.’*  Group 4: *‘I don't think it caught anyone's passion or interest. I think it just kind of was I don't know, like a filler course.’* |
|  | *Learning preferences* | Group 1: *‘EBP three has been the best… We’ve learned the most and how to actually applied it’*  *‘I don’t know if it’s changed now, but in first year, I don’t think we actually had lectures for EBP. We got the lecture notes, but we never actually went to lectures.* *[1st year online lectures]… That’s right, we just had had tutes…. And I remember everyone was complaining about it, because as first years we couldn’t do our own learning. We didn’t know how... I think that’s why when we got to second year, we actually didn’t know anything.’*  *‘I felt that when you’re in a group, you don’t get to do all the steps yourself…I didn’t actually do the search for my group … But actually having to do one on my own I found helpful’*  *Group 3: ‘…do like a systematic review in the group. By yourself is better …’*  Group 4: *‘Because we had just the lectures, I think it was more just more of an external thing in the first year…. external which is, I don't know, a bit different, compared to if you come in an listen to the lectures and you take a bit more out of it I suppose…’*  *‘I definitely learnt more in EBP two, even though pretty much it was the same.’ [face-to-face teaching]*  *‘I didn't mind EBP two* *[face-to-face teaching], I think it was just more engaging and that's why I learnt a bit more.’*  *‘EBP three, I thought was a good concept, like doing a systematic review’*  *‘I suppose I don't feel as confident as probably as other people do, because I did miss out on implementing the search strategy actually in the databases and learning properly how to do it [group project].’* |
| 3 | Learning by doing | Group 1: *‘…our systematic review though, it was helpful in the sense that I have a way better understanding of how to search and also how to critically appraise things. So when I get an article myself, I can look at it and say, “no, this is bad methodology”, stuff like that.’*  *‘I t*h*ink it [systematic review] was definitely helpful, definitely consolidated a few things.’*  *‘…this year it’s [EBP] very specific to your patients and making sure you look at patient perspective and say, “this is the evidence, but is it relevant to them?”’*  *‘EBP three has been the best… We’ve learned the most and how to actually applied it’*  Group 2: *‘I think systematic review really was good to consolidate your knowledge. So there’d be stuff like biases and how good the quality of the study is because you actually have to write that segment up and say “well this is..”, “ don’t use that poor evidence”.’*  *Q What was there in the EBP courses over the years that you thought worked well? ‘Definitely the systematic review at the end.’*  *‘…it’s made more sense to us in the last one or two years probably. The first two years we don’t really have clinical placements so we don’t see the link as much but once you start going on placement and your supervisors ask you what kind of evidence is behind this treatment you see the link.’*  *‘Yeah definitely. I think they [university staff] definitely push it, it is a big thing…Well every time you do your assignment you have to do EBP.’*  Group 3*: ‘For me it was the systematic review for my honours thesis that I actually learned it [EBP].’*  *‘Clinical placements we need to use it and apply on patients quite a lot.’*  *‘…in year three, I’ve got a lady with pain on the shoulder and on the elbow and I think on the wrist and then my supervisor told me to go home and look for complex pain syndrome…* *Yeah it definitely helped me understand the presentation and everything…’*  *‘…having that sort of assignment you learn in the lecture and then put in practice that week or put in practice and it carries through’*  Group 4: *‘I thought our placements, it [EBP] seemed quite important….All the placements wanted research…’*  *‘Sometimes to explain a treatment, for example low back pain, if you were working on TA, sometimes patients are like, "It's my back that's sore, not the front." And if you can kind of explain that evidence shows, if you work on this it fixes this and it's a good way to kind of sell the treatment to them, I guess.’*  *‘I was teaching a patient pelvic floor exercises as she was experiencing some incontinence… and I told her like evidence shows if you do this, you're less likely to get symptoms after your pregnancy, so she seems quite happy to hear about it and know that there is evidence that it works, yeah.’* |
|  | *Role models and reinforcement* | Group 1: ‘*Last placement, my tutor often referred to “new evidence in this” to his patients, and didn’t necessarily bring out a textbook but would explain it to them.’*  *‘…people are doing PD sessions within their clinics, learning new things all the time, that’s a really good way that you can implement new research and stuff like that. So in different clinics, everyone might do a bit of research and have a staff meeting every once in a while and discuss it.’*  *‘…I think it as just a shin splints and this patient came in and they thought it was their calf causing the pain, and my tutor had all this evidence on it, “no, it was this new research in this area and it was this muscle, therefore we’re going to do this”, as well as these other things that the patient had wanted to do.’*  *‘…there was a patient of a condition that my tutor hadn’t really seen before. So that required him to have to come back and book the patient in again so he could find out something, a better path to go down.’*  Group 2: *‘…on my [placement] my tutors were running like a falls class and they decided that they would do a whole evidence search about it to see if they were actually doing evidence based practice in their class. So I’ve definitely seen it a fair bit.’*  *‘I went to a hospital as well and they have once a week or once every two weeks, one person of the staff have to present a research question and the evidence behind it.’*  *‘Yeah definitely. I think they [university staff] definitely push it, it is a big thing…Well every time you do your assignment you have to do EBP.’*  Group 4:  *‘ …at X Hospital I know that they, for the area I was in, they had quite a strong focus on it. They were always bringing in “there's a lot of new research coming out”, hey were talking about that a fair bit, so I thought that was quite good.’*  *‘at the ortho department they had like an article presented every couple of weeks by one of the staff members there. That was actually pretty cool; just something that's a bit novel.’* |
| 4 | Getting the timing right | “I didn't realise …I was just like, ‘EBP just get through it’ … so maybe if I knew it was going to be such a big part, maybe I would've taken it bit more seriously”  *“…teaching how to use Endnote properly as well, ‘cause that was particularly good…”*  Group 2: *‘the library tutes were really good’*  *‘Just one library session and it changed everything for us. It was like all those times I spent hours researching for an essay, still only got a credit because my resources weren’t good and if they put that earlier on I think it would help to make it significant for you.’*  *I think especially the database searching component of EBP, like I think most of us felt like in the last EBP course, when we actually got to see someone go through a systematic search, each sort of with those exploded things and the focus and scope and all that sort of thing. It was like, “oh okay we should actually be searching a lot more thoroughly” and that was actually good to actually see that.’*  Group 3: *‘And teaching how to Endnote properly as well cause that was particularly good for honours cause it was so large, but I use it all the time now with all my assignments, but I just never knew how to use it until I started honours’* |
|  | *Optimising clinical placement experiences* | Group 1: “…would definitely appreciate it more in second year after a placement .”  Group 2: *‘…in third year when we’re starting to do a lot of our placements maybe integrating the EBP in then. Say trying to make us use it with the patients instead of just waiting till fourth year to do that because it is handy and it is really useful and I just didn’t really do much of that’*  Group 4: ‘*I had EBP right at the end in the second half of the year, so I'd finished all my placements by the time we even got shown this…’*  *‘I wish I had I known all this when I was doing [acute placement]. Research was a big part of that one.’*  *‘I had it the other way, so I did EBP in the first semester and then [acute placement], which was heaps better…So much better…I would hate to do it the other way around.’*  *‘Pretty much finished [placement] and then we found out about how to do all the searching and stuff. …Yeah, the same. …So, it would've been so good to know this two months ago’* |
|  | *Evidence searching skills needed early* | Group 2: *‘And I think as well earlier on we should have done more stuff about the search process...*  *Yes….Because that’s what it mainly focused on third year and all of us were like “oh my God.”*  *Yeah just one library session and it changed everything for us. It was like all those times I spent hours researching for an essay, still only got a credit because my resources weren’t good and if they put that earlier on I think it would help to make it significant for you.*  *Rather than all at the end...’*  Group 3: *‘And teaching how to Endnote properly as well cause that was particularly good for honours cause it was so large, but I use it all the time now with all my assignments, but I just never knew how to use it until I started honours.’*  Group 4: ‘*I think that more of a focus on that database searching would've been really good in as early as first or second year, so that we've got the rest of the course then when we're actually going onto placements and we need to find articles to help our patients and our clinical skills, we actually have that search.’* |
